# *Dr. Smartphone, can you support my trauma*? An informatics analysis study of App Store apps for trauma- and stressor-related disorders

**DOI:** 10.7717/peerj.15366

**Published:** 2023-05-09

**Authors:** Amanda Ting, Craig McLachlan

**Affiliations:** Centre for Healthy Futures, Torrens University Australia, Surry Hills, New South Wales, Australia

**Keywords:** mHealth, Mobile applications, Trauma-informed, Digital health, mTrauma

## Abstract

**Background:**

Psychological trauma is prevalent in developed countries, with prevalence rates and treatment needs exceeding health system capacity. As telemedicine and out-of-patient care are promoted, there has been an expansion of digital apps to compliment therapeutic stages in psychological trauma. To date there are no reviews that have compared these apps and their clinical utility. This study aims to identify the availability of trauma- and stressor-related mhealth apps, assess their functionality, and review their therapeutic abilities.

**Methodology:**

The authors conducted a systematic search using an iPhone 13 Pro in the Australian IOS App Store to extract trauma- and stressor-related apps that resulted from the search criteria. A cross-adaptation of the *Mobile App Rating Scale* (MARS) and the *Comprehensive App Evaluation Model* (CAEM) were used as a framework to produce the *mTrauma App Evaluation Conceptual Model* and *Informatics Framework*. App content descriptors were analysed based on their general characteristics, usability, therapeutic focus, clinical utility, data integration. Following an applicability in concordance with psychological trauma-informed delivery.

**Results:**

A total of 234 apps resulting from the search strategy were screened, with 81 apps that met the inclusion criteria. The majority of apps were marketed to 4+ to 17+ years of age, categorised as ‘health and fitness’, with the highest target markets observed for adolescents, children, parents, clinicians, and clients. A total of 43 apps (53.1%) contained a trauma-informed specified section, and 37 (45.7%) incorporated a section useful to support trauma-related symptoms. A significant number of apps there was an absence of therapeutic utility (in 32 apps (39.5%)). Most apps were supporting post-traumatic stress disorder-informed, cognitive behavioural therapy and eye movement desensitisation and reprocessing. Provision of psychoeducation, courses, guided sessions, trainings, self-reflection/journaling, symptom management and progress tracking were highly present.

**Conclusions:**

Trauma-informed mobile apps are available in the App Store, expanding in its target market reach and usability, with an increase of creative psychotherapies being introduced alongside conventional modalities. However, based on the app descriptors, the scarcity of evidenced-based testimonials and therapeutic applicability remains questionable for clinical validity. Although mhealth tools are marketed as trauma-related, current available apps employ a multifunctional approach to general psychological symptomatology, through to associated comorbid conditions and emphasizes on passive activity. For higher uptake on user engagement, clinical application and validity, trauma-apps require curated specification to fulfil its role as complimentary psychological treatment.

## Introduction

Psychological trauma can be defined as one or more events that, due to their characteristics, that can alter the subject’s self-perception, memories, and mental health (Perrotta, 2019). Complex psychological trauma is more intricate and pervasive than an individual traumatic incident which generally occurs during developmental phases compromising childhood development (Singh et al., 2021). When this occurs early trauma can result in subtle changes to brain structure and function leading to attentional and behavioral disorders that can persist into adulthood.

Psychological trauma, its aftermath and its implications pose great concern for public mental health. Millions of people worldwide are psychologically affected by traumatic stress (Kleber, 2019). Exposure to traumatic events has negative effects on physical and mental wellbeing (Benjet et al., 2016). Any experience that exposes a person to sexual assault, major injury, natural disasters, childhood trauma, discrimination or the prospect of death has the potential to be traumatic (Williams, Printz & DeLapp, 2018; Dye, 2018; Guina et al., 2018; Elder et al., 2017; García et al., 2015; Kirkinis et al., 2021; Kucharska, 2018). Trauma can result from exceptional circumstances, such as the COVID-19 pandemic (Kim, Nyengerai & Mendenhall, 2022). Recent studies suggest that those impacted are prone to develop traumatic stress reactions linked to future anxiety, viral exposure, and stressful socioeconomic situations (such as unemployment, isolation, illness, death, and grief) (Bridgland et al., 2021; Łaskawiec et al., 2022; García-Fernández et al., 2022). The impending global mental health implications were challenged by increased hospitalizations, post-COVID-19’s ‘great resignation’ movement (including mental health providers) and burnout (Jakovljevic et al., 2020). Following the effects of the ongoing COVID pandemic, the 21st century mass-witnessed and experienced the Russian-Ukrainian crisis and its bi-directional effects including refugee displacement, rise in living costs due to the economic multiplier effect globally, and the generational impact of the conflict on mental health globally due to online media (Patel & Erickson, 2022; Cai et al., 2022; Chaaya et al., 2022; Jawaid, Gomolka & Timmer, 2022; Riad et al., 2022). These examples above, suggest psychological trauma has significantly increased in prevalence, placing significant strain on current health systems, as they cannot meet delivery needs. Henceforth there is an increased need for mental health tools, such as trauma-mhealth apps for urgent need cases that cannot find support with traditional health service pathways.

Exposure to traumatic backgrounds can lead to experiences of varied developmental trajectories for trauma- and stressor-related symptoms and illnesses. Major catastrophe characteristics vary, and several pre-, peri-, and post-disaster societal and personal variables affect the resulting mental health of those affected, including PTSD, depression, anxiety, drug and alcohol abuse, suicidality, and decrease in wellbeing (Moustafa et al., 2021; Sakuma et al., 2020). Studies have concurred the psychological burdens of complex trauma or cumulative trauma extensively affects attachment, social functioning, and increased comorbidities such as obsessive-compulsive disorder and schizophrenia (Osland, Arnold & Pringsheim, 2018; Gabínio et al., 2018; Nichter et al., 2019). Trauma trajectories differ based on sub-syndrome symptomatology (elevated symptoms below the diagnostic threshold), delayed-onset symptomatology (elevations above the diagnostic threshold that emerge after a significant delay), and minimal-impact resilience (stable psychological and physical health from before to after the potential traumatic events) (Galatzer-Levy, Huang & Bonanno, 2018). As populations or cohorts may greatly overlap at any given time, cross-sectional diagnostic categorization may neglect or conflate separate trajectories, highlighting the complexities of trauma- and stressor-disorder diagnosis and prognosis.

There is significant individual variation in how pathological stress may lead to trauma. Additionally, how the individual reacts to psychological therapies is significantly variable. Although trauma-focused cognitive behavioral therapy (CBT-T) and eye-movement desensitization and reprocessing (EMDR) have been heavily adopted conventionally (Ford, 2021; Rodenburg et al., 2009; Gelinas, 2003), studies outlined the adoption of newer psychotherapies such as somatic body therapies and creative arts modalities have beneficial outcomes in treating trauma (Baker et al., 2018). For instance, treating PTSD offers opportunities for the creation of therapies that take individual-level characteristics into account when determining treatment response and supports the diverse trajectories of psychotherapy responses (Dewar, Paradis & Fortin, 2020). Aside from recommendations for the psychological treatment of trauma, additional importance in the progress of treatment should prominently reflect the therapy dyad, responsiveness, and flexibility of adjusting treatment (Norcross & Wampold, 2019).

As telemedicine and outpatient care become more common practice, mHealth is complementing the way healthcare may be provided. The advantages of integrating mHealth applications include patient segmentation strategies and service customization in a way that is meant to be patient-centered to match the requirements of the individual (Paglialonga et al., 2019). Considering the global scarcity of mental health clinicians, rapid innovations of mobile health tools present considerable potential towards seamless patient care, early interventions, real-time facilitation of information and accessibility to undersupplied population groups (Tal & Torous, 2017; Messner et al., 2019; Baños, Herrero & Vara, 2022; Marshall, Dunstan & Bartik, 2020). On the contrary, the effectiveness and dangers of self-driven mobile applications have frequently been questioned by health experts (Balapour et al., 2019; Dahlhausen et al., 2021; Tarricone et al., 2021; Della Vecchia et al., 2022). Making the decision to use an app with a patient differs somewhat from making the decision to use a certain type of psychotherapy or medicine, as mental health professionals are not necessarily well-informed across various technological m-health platforms (American Psychiatric Association (APA), 2022). Examining the clinical utility of m-health solutions include evidence for therapeutic validity, prior clinical testing or trials, consumer safety, harmful content, data sharing and privacy concerns (Anthes, 2016; Akbar, Coiera & Magrabi, 2020; Huckvale et al., 2020). Apps as ‘digital clinics’ have the prospective to support individuals through interactive tools that aid in treatment adherence, but if they are inaccurate and unreliable, they pose significant psychophysiological risks primarily if users adopt information solely from apps to make crucial decisions about their health.

The use of mobile health (mHealth) has never been more ubiquitous due to the number of smartphone users and ease of access they subsequently provide. Despite its advantages or pitfalls, downloadable apps that are both interactive and psycho-educational are widely accessible to address a growing number of mental health conditions. The effectiveness of mobile app-based therapies and management for mental health has been validated by prior research and empirical investigations (Donker et al., 2013; Lippman, 2013; Watts et al., 2013; Rathbone & Prescott, 2017). Wang, Varma & Prosperi (2018) reviewed mobile apps for managing mental health disorders for people of all ages, including depression, anxiety, bipolar disorder, psychosis, post-traumatic stress, substance use disorders, sleep disorders, and suicidal behavior, where they emphasized that the majority of the apps lacked adequate clinical validation. The various widely accessible mHealth apps for mental health conditions such as depression, anxiety, suicide prevention, and addiction, trauma-centered mobile apps are lacking both in availability and support by evidenced based research.

Applications concentrating on psychological trauma appear to be more targeting post-traumatic stress disorder. Only one trauma-related app (PTSD-Coach) was featured among well-known mental health apps in a van Ameringen et al. (2017) assessment of mobile applications for obsessive compulsive disorder, anxiety, and mood disorders that emphasized having potential for therapeutic value. On the other hand, Goreis et al. (2020) conducted a meta-analysis on the efficacy of self-management-based apps for PSTD symptoms in populations with sub-threshold PTSD symptoms or PTSD and concurred the two groups (app-based *vs*. waitlist control groups) did not differ significantly regarding post-treatment in PTSD or depressive symptoms. In another comprehensive assessment of 69 high-quality PTSD mobile applications that were evaluated in accordance with psychological treatment and self-help approaches, Sander et al. (2020) concluded that only one app had been evaluated in a randomized controlled study. This highlights the absence of scientific evidence of mHealth applications committed to addressing the wider scope of trauma symptomatology. Thus, the significance of this review will highlight the literature gap pertaining to the landscape of trauma-focused mental health apps.

This study has three aims: (1) to identify the availability of trauma- and stressor-related mhealth apps available through the ioS iPhone App Store (Australia) using a descriptive informatics approach, and (2) to assess the tools and functionality of mhealth trauma-related apps based on their psychological foundations embedded within app descriptors and (3) critically argue evidence for therapeutic integration.

## Materials and Methods

### Search strategy and inclusion criteria

We conducted a search on an iPhone 13 Pro to systematically screen the IOS App Store on 4th June 2022 using the following search key terms: ‘trauma’, ‘trauma mental health’, ‘traumatic stress’, ‘trauma management’, ‘trauma recovery’, ‘post-traumatic stress’, ‘PTSD’ and ‘moral injury’.

Applications (apps) were identified if their title, subheading, or description indicated: (1) they were contextualized for mental health, (2) available in English, and (3) officially accessible for download in Australia. Apps were eligible for inclusion if they: (a) focused on trauma- and stressor-related symptomatology/disorders (in concordance with the DSM-V), (b) contained a specific section with trauma-informed care or (c) included a section that is useful to support prognostics condition relative to trauma experiences (*e.g*., mood, anger, sleep). The authors’ terminology of ‘section’ is described as a tab, window with psychoeducation, tool, activity, guided trainings, or other functions within the app’s platform that a user can engage with.

### Mobile app evaluation framework

Author 1, a licensed psychotherapist (provisioned by PACFA) acquired and synthesizes the data extracted from included apps using a cross-adaptation of the Mobile App Rating Scale (MARS) (Stoyanov et al., 2015) and the Comprehensive App Evaluation Model (American Psychiatric Association (APA), 2022).

### Mobile app rating scale

MARS is a straightforward, unbiased technique for categorizing and rating mHealth apps. It may also serve as a checklist for the creation of new released, high-quality health apps. The uMARS provides capability to extract rich information from target users concerning mobile apps through a 20-item measure with four objective subscales (Stoyanov et al., 2016). Both MARS and uMARS models were used. Six items from two subscales were extracted for adaptation: Subscale 1. Engagement (items: customization, interactivity, and target group). Subscale 2. Information (items: quality of information, and quantity of information). Included subscale adaptions were utilized to frame this study that best structures our descriptive informatics analysis. Our study’s first aim is to provide an overview of trauma-apps using information analytics as to how apps are presented to target audiences. Selected apps were thoroughly screened and did not require downloads. Thus, reliability testing for efficacy and quality were not incorporated.

### App evaluation model

The Comprehensive App Evaluation Model was developed by the APA App Advisor, an American Psychiatric Association (APA) initiative to assess the expanding utility of mHealth technologies. Their evaluation process objective is to use a hierarchical rating system and embedded rubric to help APA members, patients, and other providers understand key information that should be reviewed when selecting an app and how this differs from selecting more conventional therapeutic interventions (American Psychiatric Association (APA), 2022). The model posits significant factors for consideration upon user engagement through critically assessing the app’s ‘accessibility, privacy and security, clinical foundation, engagement and interoperability’ (Lagan et al., 2020). Selecting the most appropriate app based on clients’ presenting issues should lead to better clinical decision-making and better patient outcomes. Since this model is an adaptable tool (Lagan et al., 2021), we have contextualized the following four steps (out of five) to produce specified trauma-related evaluative inquiry: (1) Access & background, (2), Clinical Foundation, (3) Usability, and (4) Therapeutic Goal.

### mTrauma app evaluation conceptual model

**Figure 1:**
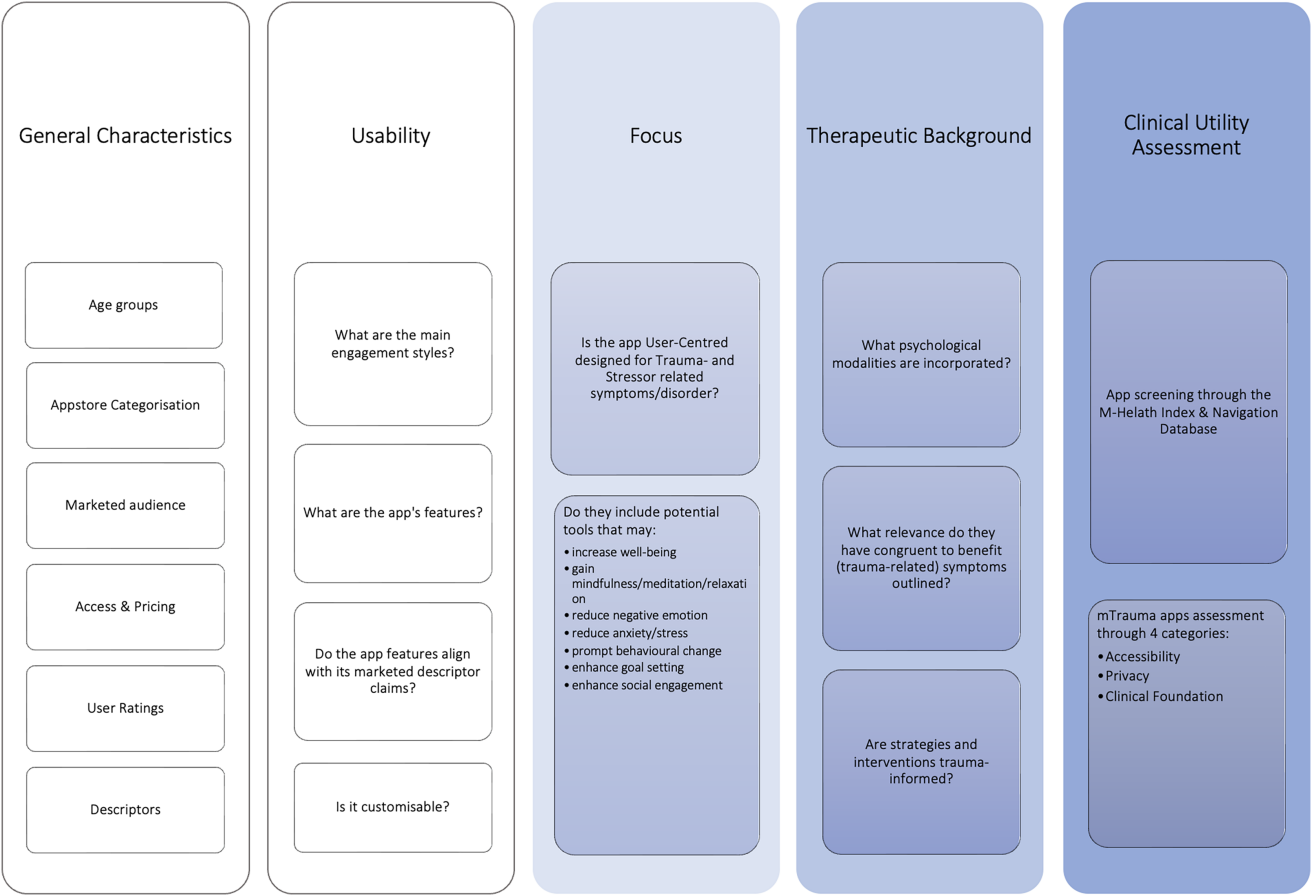
Ting & McLachlan (2022) mTrauma app evaluation framework.

The mtrauma app evaluation conceptual model was proposed that guided the structure of this informatics study. Outlined in grayscale in Fig. 1 displays the cross-adapted elements that we have developed from the MARS and CAEM frameworks, followed by sections curated for this study outlined in blue.

Both ‘general characteristics’ and ‘usability’ tabs were incorporated to appease the study’s 1st aim. The degree to which people can receive, process, comprehend, and convey health-related information necessary to make wise health decisions is referred to as health literacy, which is an important determinant of health (Sørensen et al., 2012; Berkman, Davis & McCormack, 2010). When people utilize the Internet and mobile devices to seek health information, they are demonstrating their eHealth literacy (Lin & Bautista, 2017), according to the term used by Norman & Skinner (2006). To answer our predefined aim 1, this requires a descriptive narrative analysis of individual app characteristics, such as, identifying general characteristics that inform how trauma-related apps relay health-related information through descriptors, marketing intentions (age groups, App Store categorization, audience, access, and pricing).

The use of mobile phone health applications may appear to be widespread at this point, but much is still unknown. There is a lack of data on key parameters, such as the population’s usage of health applications, their adoption or non-adoption, and their reasons for discontinuing use (Krebs & Duncan, 2015). Usability is characterized as the efficacy, efficiency, and satisfaction with which users accomplish goals in a given context of usage (Islam et al., 2020). Determining an app’s usability is a crucial component of successful mHealth app development and uptake, just like it is for other digital applications (Zapata et al., 2015, 2018; Maramba, Chatterjee & Newman, 2019). In relation to uptake and user engagement, four criteria were posited (engagement styles, app features, app alignment with its marketed descriptor and customization) in determining the usability of trauma-related apps.

The third category (focus) were constructed specified accordingly to trauma- and stressor disorder-related evaluation and addresses aim 2. Specifically, to assess the app’s tools and functionality. Exploring the apps’ therapeutic background is followed by our 3^rd^ aim, to discuss their potential therapeutic qualities.

Many current mHealth treatments may not be as successful as those that engage end-users in the design process as they are often based on the structures of the present healthcare system. To be user-friendly and seen as beneficial, apps must be created with sufficient consideration for its intended users (Schnall et al., 2016). The Information Systems Framework (ISR) has been suggested as a practical method for designing a mobile app that encompasses end-user design preferences. The design process involves three cycles (the relevance, design, and rigor cycles). Supporting distinct user-centered development, Farao et al. (2020) suggested merging the ISR framework with design thinking in the development of mHealth based interventions. Considering this, the third criterion considers if the app user-centered design for trauma- and stressor-related symptoms/disorder, and if they include tools that support trauma symptomatology.

The proliferation of evidence-based or non-evidenced based mental health applications are not governed by a universal benchmark. Many of the mHealth applications that are currently on the market are lacking capabilities that would significantly increase their functionality. In addition, experimental trial-based validation of apps is rarely conducted or published by app developers (Bakker et al., 2016). Additionally, “marketplace interposition” is a duty placed upon developers. This phrase describes the encouragement of self-treatment and unlicensed medical practice by society as a result of technology improvement (Chatzipavlou, Christoforidou & Vlachopoulou, 2016). Clinical research has shown certain applications to have positive effects, but many, if not most, have not. Although app developers may make statements about the clinical efficacy or history of their applications using adjectives like “trauma-informed” or “led by clinical insights,” there is frequently no data to support these assertions. The ideal for criterion four investigates the psychological modalities incorporated, their relevance, if incongruent to benefit trauma symptomatology, and if strategies and/or interventions are trauma-informed in comparison to clinical literature.

The screening of clinical utility and data integration for therapeutic adoption is the final phase in our model. As iterated by the APA in their CAEMS model, apps should not fragment treatment (American Psychiatric Association (APA), 2022). The client (or patient) and practitioner should have the ability to exchange and discuss data or obtain feedback from the app as necessary (or at least have the choice to do so). Thus, data integration within the therapeutic dyad becomes crucial in this paradigm. We have considered if apps have a privacy policy, therapeutic foundation and data integration that can be used in collaboration with a provider/practitioner in their suitability for adoption from a clinical standpoint. In arguing the trustworthiness of trauma-related apps assisting the clinical process through a descriptive approach, apps were screened through the M-Health Index and Navigation Database (MIND, https://mindapps.org/FrameworkQuestions) with areas contextualized to inform on trauma-specified disorders.

### M-health index & navigation database

The framework was created by Lagan et al. (2020) in conjunction with the American Psychiatric Association’s (APA) app evaluation model to reflect consensus from various stakeholders, including mental health professionals, social workers, data scientists and everyday users (Spadaro et al., 2022). Objective questions based on the APA App Evaluation Model, which presents Accessibility, Privacy & Security, Clinical Foundation, Engagement Style, and Interoperability as primary areas to consider, are used to inform each app’s entry in the database. The purpose of this database is to provide users (both clients and clinicians) with the knowledge necessary to make decisions based on the app qualities that are most important to them, while considering the selection of an app is a personal choice based on many different individual circumstances. Since this study provides a descriptive account of evaluation without proceeding to download all apps, and authors were unable to formulate a homogenous data set to fulfil all criteria due to the limited information provided by tech developers, the results from this fifth phase included only applications that were registered on the MIND database at the time of study.

### General characteristics

The following classifications were included to capture descriptive information of screened apps: (1) Age groups, App Store Categories, intended market, availability to download for ‘free’, required payment and fee structure, user ratings and app descriptors (sub-headings & descriptions). Ratings were ranked (1 to 5 stars) and analyzed provided they sufficed a minimum of three user ratings. When app descriptions were insufficient, further information were extracted through web-searches (*e.g*., using the app name, tech developer homepage when present).

### Therapeutic background

Four sub-categories were outlined to capture the apps’ therapeutic landscape: (1) Specified for trauma- and stressor-related symptomatology/disorders, (2) Contains a section specified for trauma-informed care, (3) Contains a section that is useful to support trauma-related symptoms according to the app’s description and lastly, (4) non-specified therapeutic background mentioned.

### Provision of technique and tools

The presence of the following elements was investigated to outline the apps’ provisional capabilities: (1) Psychoeducation, (2) self-reflection or journaling, (3) clinical assessment, (4) symptom management and progress tracking, (5) Imbedded courses, trainings, guided sessions, and activities, (6) tips, advice, and resources, (7) links to services, access to clinicians and referrals, (8) option for user customization and other miscellaneous options.

### Assessment for therapeutic utility

To assess utility and integration, our quality rating utilized three categories with a collective total of eight criteria from the MIND framework to examine trauma-related apps: Category (1) Accessibility, Category (2) Privacy, and Category (3) Clinical Foundation. From the three categories, eight criteria were defined that permitted a total score to be provided for each app. The definitions of these criteria are in Table 1. The findings from each criterion were extracted from the MIND website allowing for assessment of clinical utility and integration. It should be noted that Criterion 7 and 8 have been adapted to identify the app specified trauma and stressor-disorders, symptoms and/or comorbidities.

**Table 1:** Assessment of apps criteria guide.

Category	Criteria	Rating guide
Accessibility	1. Can the app be accessed offline?	Accessibility of mHealth apps is essential to usability and user engagement. Considerations include offline accessibility to app features, data proprietorship, and sharing capabilities to external parties (*e.g*., caregivers, providers).
	2. Who owns the data?	
	3. Can the data be exported?	
Privacy	4. Does the app have a privacy policy?	Users can study privacy policy prior to installation to gain a clear understanding of what personal data the app will access and where data is stored.
	5. Where is the app data stored?	
Clinical foundation	6. Does the app have relevant content?	Users are guided by the apps’ therapeutic foundations and content in app engagement displays relevance to its marketing claims.
	7. Does the app do what it claims?	
	8. Does the app specify conditions supported in relation to trauma and stressor-disorders/comorbidities/symptoms? If so, what are the conditions specified?	

## Results

### Search

**Figure 2:**
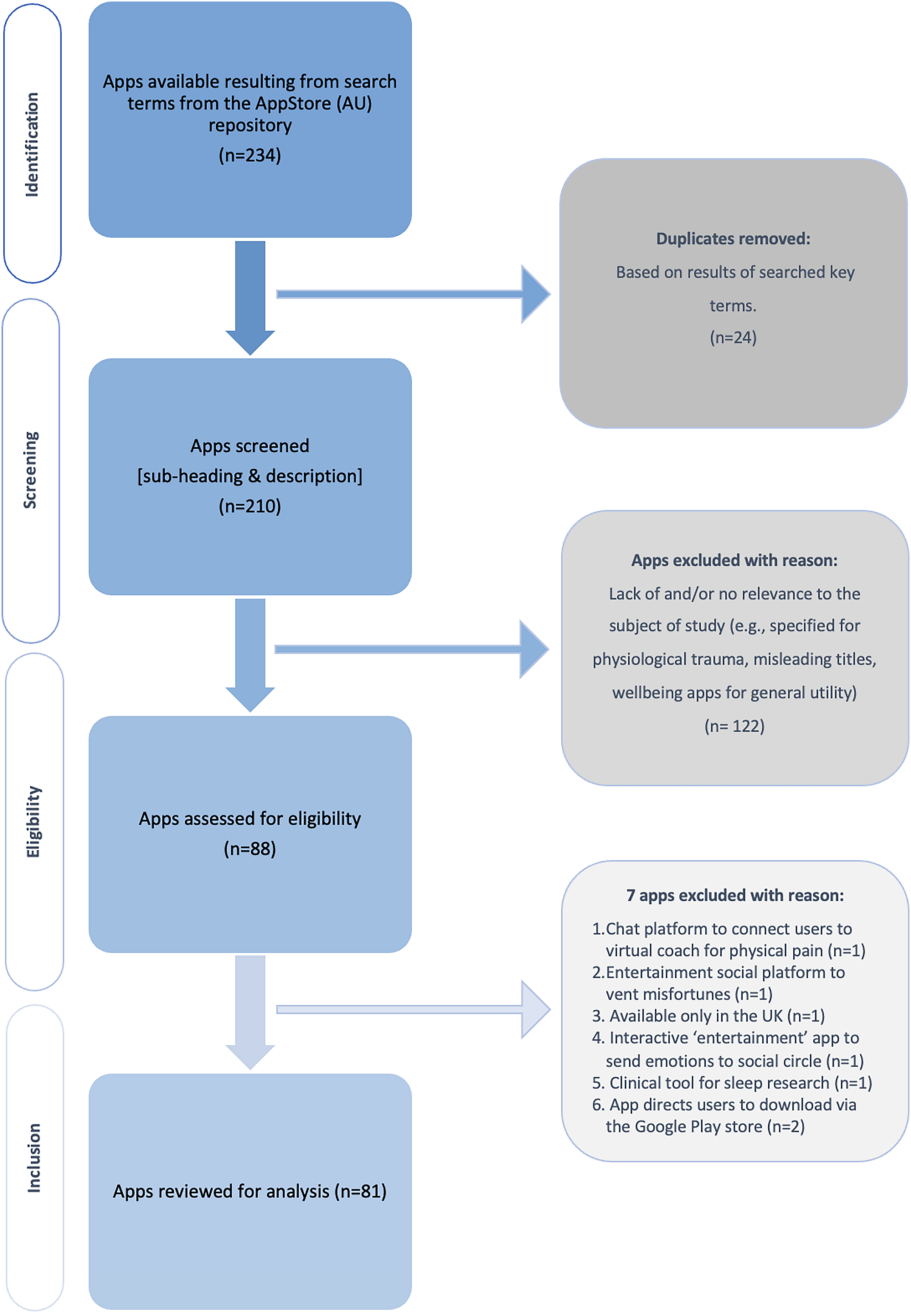
Flowchart of app inclusion.

A total of 234 apps resulted through the authors’ cumulative search strategy with 88 apps (37.6%) assessed for eligibility. A total of 81 Apps (34.6%) met the study’s inclusion criteria and analyzed. Figure 2 illustrates the staging process of inclusion.

### General characteristics

Table 2 provides a reviewed compilation of trauma-related mHealth apps’ characteristics from the Australian iPhone App Store presented in detail. Age groups indicated for safe app usage spanned 4+ to 17-years and above. There is a contrasting disparity with the highest number of apps marketed for 4+ (*n* = 34, 42%) compared to only one app (1.2%) for 9+ audiences. There were distributions across six categories with *Health & Fitness* (*n* = 56, 69.1%) as the most frequented classification used by developers, as expected for mHealth apps. We observed apps were marketed to three main target markets which were: (1) Adolescents, children, parents, and caregivers, (2) clinicians and clients, and (3) veterans, military personnel, and soldiers. Majority of apps were free-of-charge (*n* = 72, 88.9%). The cost of 11 paid apps (13.6%) ranged from AUD $1.49 to $14.99. The highest user ratings revolved around the 4-star range (*n* = 16, 19.8%) but were three times significantly over-shadowed by apps that were not rated or not applicable for ratings (*n* = 49, 60.5%). Although our initial analysis included apps awarded across 1* to 5*, results were displayed accordingly to the minimum three ratings requirement.

**Table 2:** General characteristics of trauma-apps. The Apple App Store uses app ratings in the measurement of ‘star(s)’ that determines users’ feedback or experience of an app. Users can award an app a rating between 1 and 5, with 5 being the highest. We have utilised the asterisk (*) as a symbol to indicate a star. Not applicable (N/A) indicates apps that have yet to receive any ratings on the App Store. Apps with a minimum of three user ratings include apps with 2, 2 and 3 star ratings combined.

General characteristics	N	(%)
**Age groups**		
17+	15	18.5%
12+	31	38.3%
9+	1	1.2%
4+	35	43.2%
**Category**		
Casual	1	1.2%
Education	4	4.9%
Health & fitness	57	70.4%
Lifestyle	6	7.4%
Medical	15	18.5%
Social networking	1	1.2%
**Target market**		
Adolescents, children, parents & caregivers	9	11.1%
Clinicians and clients	9	11.1%
Couples & partners	1	1.2%
First responders, medical care personnel, police, and public safety professionals	8	9.9%
Support community	6	7.4%
Veterans, military combat personnel, or soldiers (including families of)	7	8.6%
Sexually abused	1	1.2%
Mothers	1	1.2%
**Availability and payment**		
Free	72	88.9%
Paid (AUD)	11	13.6%
$1.49	1	1.2%
$4.49	1	1.2%
$7.99	5	6.2%
$9.99	2	2.5%
$10.99	1	1.2%
$14.99	1	1.2%
**User ratings**		
N/A	49	60.5%
Apps with a minimum of three user ratings	24	29.6%
5*	6	7.4%
4* (4–4.9*)	16	19.8%
3*	3	3.7%

### Therapeutic background

The following four sub-categories provides a synopsis that formed the apps’ therapeutic landscape detailed in Table 3. More than half of the included app (*n* = 48, 59.3%) descriptors recommended they were curated for trauma- and stressor-related symptoms and disorders. Similarly, 53.1% (*n* = 43) of apps embodied a trauma-informed section, with three clinically established trauma-informed evidenced-based modalities forming the most common therapeutic backgrounds: Post-traumatic stress disorder informed strategies and interventions (*n* = 9, 11.1%), Cognitive Behavioral Therapy (*n* = 8, 9.9%), and Eye Movement Desensitization and Reprocessing (*n* = 7, 8.6%). Other trauma-specified sections were statistically homogenous which included therapies such as Acceptance & Commitment, Cognitive Processing, CBT-Insomnia (CBT-I), Exposure therapy, psychological first aid and trauma-informed care and two assessments. Thirty-seven apps (45.7%) incorporated a section deemed useful to support trauma-related symptoms with journal (self-reflection) and mood tracking deemed the highest component present (*n* = 14, 17.3%). Five apps proposed holistic and somatic elements including meditation, mindfulness, tension and trauma release techniques and yoga. The remaining apps included art therapy, narrative therapy, sensory and imagine identification, platforms to engage with online communities and organizational-based models. A total of 32 app (39.5%) descriptors did not specify a therapeutic foundation or framework.

**Table 3:** Therapeutic background and sub-categories. Table 3 outlines the therapeutic backgrounds of the applications, which are divided into four subcategories (indicated in bold): 1. Apps that list at least one (or more) symptomatologies or disorders associated with trauma and stress. 2. Apps that include a specified section dedicated to provide trauma-informed treatment are included in this sub-category, which has 14 components. 3. Eleven therapeutic modalities and/or techniques are defined in the apps that, according to their descriptions, offer a section that could be helpful to support symptoms associated to trauma and stress disorders. And, 4. Apps with no specified therapeutic background and/or model.

Therapeutic background & sub-categories	N	(%)
**Specifically developed for trauma- and stressor-related symptomatology/disorders**	**48**	**59.3%**
**Contains a section specified for trauma-informed care**	**43**	**53.1%**
Adverse Childhood Experiences (ACE’s) Questionnaire	1	1.2%
Acceptance & Commitment Therapy (ACT)	2	2.5%
Cognitive Behavioral Therapy (CBT)	8	9.9%
Cognitive Behavioral Therapy for Insomnia (CBT-i)	1	1.2%
Cognitive Processing Therapy (CPT)	2	2.5%
Dialectic Behavioral Therapy (DBT)	1	1.2%
Emotional Freedom Therapy-Tapping (EFT-T)	3	3.7%
Eye Movement Desensitization and Reprocessing (EMDR)	7	8.6%
Post-Traumatic Stress Disorder (PTSD) informed strategies & interventions	9	11.1%
Exposure Therapy (ET, and Prolonged Exposure therapy–PE)	2	2.5%
Psychological First Aid (PFA)	1	1.2%
Trauma-informed care	2	2.5%
Trauma-informed neurobiology/neuroscience	2	2.5%
PCL test	2	2.5%
**Contains a section useful to support trauma-related symptoms according to app description**	**37**	**45.7%**
Art therapy	2	2.5%
Journal & mood tracking	14	17.3%
Meditation	1	1.2%
Mindfulness	2	2.5%
Narrative therapy	1	1.2%
Online community	6	7.4%
Organization-based frameworks (TRiM Military, TRiM Police, Community Wellness model & STAIR)	4	4.9%
Positive psychology	4	4.9%
Sensory & image identification	1	1.2%
Tension & trauma release technique	1	1.2%
Yoga therapy (trauma-healing)	1	1.2%
**Not specified therapeutic background and/or model**	**32**	**39.5%**

### Provision of technique and tools

Over half of the total included apps included a section on psychoeducation (*n* = 58, 71.6%), provided courses, trainings, guided sessions, and activities as user content (*n* = 56, 69.1%) (detailed in Table 4). Thirty-seven apps (45.7%) included tools for users to manage their symptoms and track their progress, whereby results could be ‘saved, printed or stored’. Thirty apps (37%) provided tips, advice, and resources. Self-reflecting tools that guide cognitive restructuring, processing, and meaning making were observed in twenty-four (29.6%) apps through passive journaling or app-embedded AI algorithm-generated prompts. Other features included links to support or emergency services, ‘click’ to view referrals (*e.g*., list of therapists generated based on app partnerships) and access to mental health providers (*n* = 21, 25.9%) and eighteen apps (22.2%) were designed to offer users some form of customization (*e.g*., settings reminders, compiling periodic results). Only twelve apps (14.8%) included a form of self-administered clinical assessment but did not explicitly provide sufficient detail within descriptors whether results were provided. Thirty-one apps (38.3%) marketed both ‘special’ features and additional ‘content’ were highlighted within their descriptors. ‘Novel’ features to attract users included undisrupted ad-free engagement, exclusive access to events or workshops, option to ‘share’ results with clinicians seamlessly, in-app link to social media or community platforms, one ‘click’ to EAP, personalized settings, and face recognition for enhanced-privacy protection. Additional features, whether observed as less attractive to some users, were subscription-based pricing tiers (often after the free-trial ends), required a login or account creation.

**Table 4:** App provision of technique and tools.

Provision of technique & tools	N	(%)
Psychoeducation	58	71.6%
Self-reflection/journaling	24	29.6%
Clinical assessment	12	14.8%
Symptom management/progress tracking	37	45.7%
Courses/trainings/guided sessions/activities	56	69.1%
Tips/advice/resources	30	37.0%
Link to services/access to clinicians/referrals	21	25.9%
Option for in-app customization	18	22.2%
**Others**	31	38.3%
(*E.g*., Ad-free, requires an account, access to events, subscription-based, reminders, option to ‘share’ results delivered to clinicians, links to social media, in-app link to EAP, face-recognition)		

### App assessment for therapeutic utility registered on the MIND database

**Figure 3:**
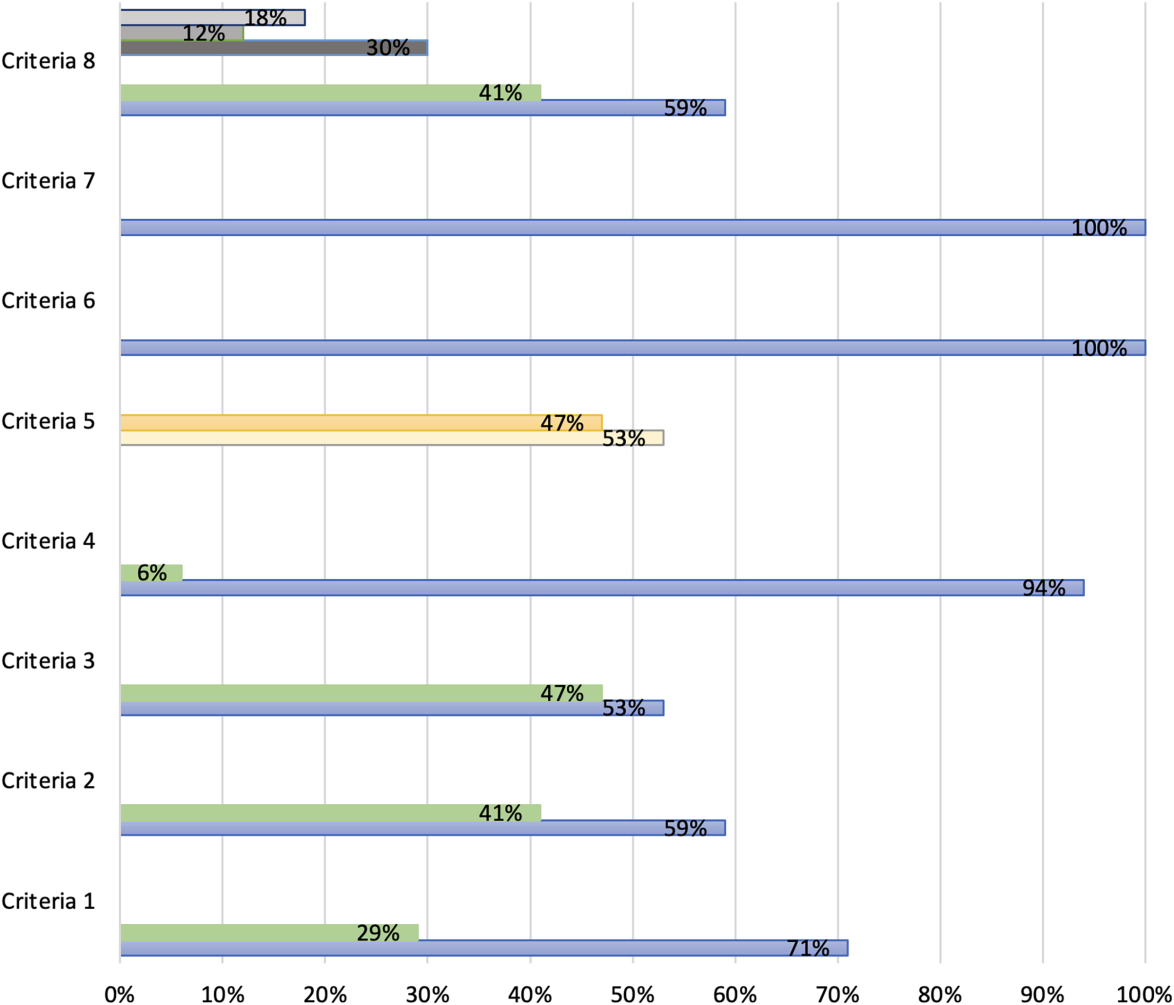
Assessment of trauma-apps registered on the MIND database across eight criteria.

A total of 81 apps used in our study were inserted in the search engine tool on the *MIND* website. As a result, only 17 apps (21%) were registered on the platform and eligible for screening (*detailed in Table 5). A total of 12 apps (71%) were accessible offline, users owned their data for 10 applications (59%) and nine apps (53%) allowed data to be exported. A high number of apps displayed a transparent privacy policy (*n* = 16, 94%) with only a slight difference between data stored on users’ device (*n* = 9, 53%) and servers (*n* = 8, 47%). Under clinical foundation, criteria 6 and 7 resulted all 17 apps have relevant content and function that align to usability claims. Despite the significance of its preceding criteria, only 10 apps (59%) specified a condition within trauma and stressor-related disorders and/or comorbidities. Finally, an overall app quality rating was calculated by summing the number of criteria met out of a possible maximum score of eight (detailed in Fig. 3). Each criteria was quantified by responses on the basis of a binary response ‘yes’ or “no”. Only criteria 5 was an exception, with apps that stored data on ‘device’ included as a quality score. The mean across all eight criteria for the 17 apps was 0.75 +/−0.244 (SD).

**Table 5:** Screening of trauma-related apps registered on the MIND database.

App name	Accessibility			Privacy				Clinical foundation	
	Criteria 1	Criteria 2	Criteria 3	Criteria 4	Criteria 5	Criteria 6	Criteria 7	Criteria 8	
	Can the app be accessed offline?	Do users own the data?	Can data be exported?	Does the app have a privacy policy?	Where is the app data stored?	Does the app have relevant content?	Does what it claims?	Does the app specify a condition within trauma and stressor- disorders/comorbidities	Condition specified
ACT coach	Yes	Yes	Yes	Yes	Device	Yes	Yes	Yes	PTSD
Bloom: CBT therapy & self-care	Yes	Yes	Not specified	Yes	Server	Yes	Yes	Not specified	Not specified
CBT thought diary	Yes	Not specified	Yes	Yes	Server	Yes	Yes	Yes	Mood disorders, stress & anxiety
CPT coach	Yes	Yes	Yes	Yes	Device	Yes	Yes	Yes	PTSD
iChill	Yes	Not specified	Not specified	Yes	Server	Yes	Yes	Yes	Stress & anxiety
Insomnia coach	Yes	Not specified	Yes	Yes	Device	Yes	Yes	Yes	Sleep, stress & anxiety
Meomind—free therapy	Not specified	Not specified	Not specified	Yes	Server	Yes	Yes	Not specified	Not specified
Mission reconnect	Not specified	Not specified	Not specified	Yes	Device	Yes	Yes	Not specified	Not specified
Moodnotes—mood tracker	Yes	Yes	Yes	Yes	Device	Yes	Yes	Not specified	Not specified
Mood balance—mental health	Yes	Yes	Not specified	Yes	Server	Yes	Yes	Not specified	Not specified
PE coach not specified	Yes	Yes	Yes	Yes	Device	Yes	Yes	Yes	PTSD
PFA mobile	Yes	Yes	Not specified	Yes	Device	Yes	Yes	Not specified	Not Specified
PTSD coach	Yes	Yes	Yes	Yes	Device	Yes	Yes	Yes	PTSD
PTSD family coach	Yes	Yes	Yes	Yes	Device	Yes	Yes	Yes	PTSD
Reflectly—journal & AI diary	Not specified	Yes	Yes	Yes	Server	Yes	Yes	Not specified	Not specified
Sleep restore	Not specified	Not specified	Not specified	Yes	Server	Yes	Yes	Yes	Sleep & PTSD
Stress less TRE	Not specified	Not specified	Not specified	Not specified	Server	Yes	Yes	Yes	Stress, anxiety & PTSD

## Discussion

Our article acknowledges Sander et al.’s (2020) application of the MARS rating scale and assessment for rating PTSD-based mobile apps. We have used a cross-adaptation of *MARS* and the *Comprehensive App Evaluation Model* to develop a trauma-specified app evaluation conceptual framework which formed the basis for our informatics study. Our study screened all available trauma- and stressor-related mhealth apps that resulted from key search terms accessed *via* the Australian ioS iPhone App Store in June 2022.

**Figure 4:**
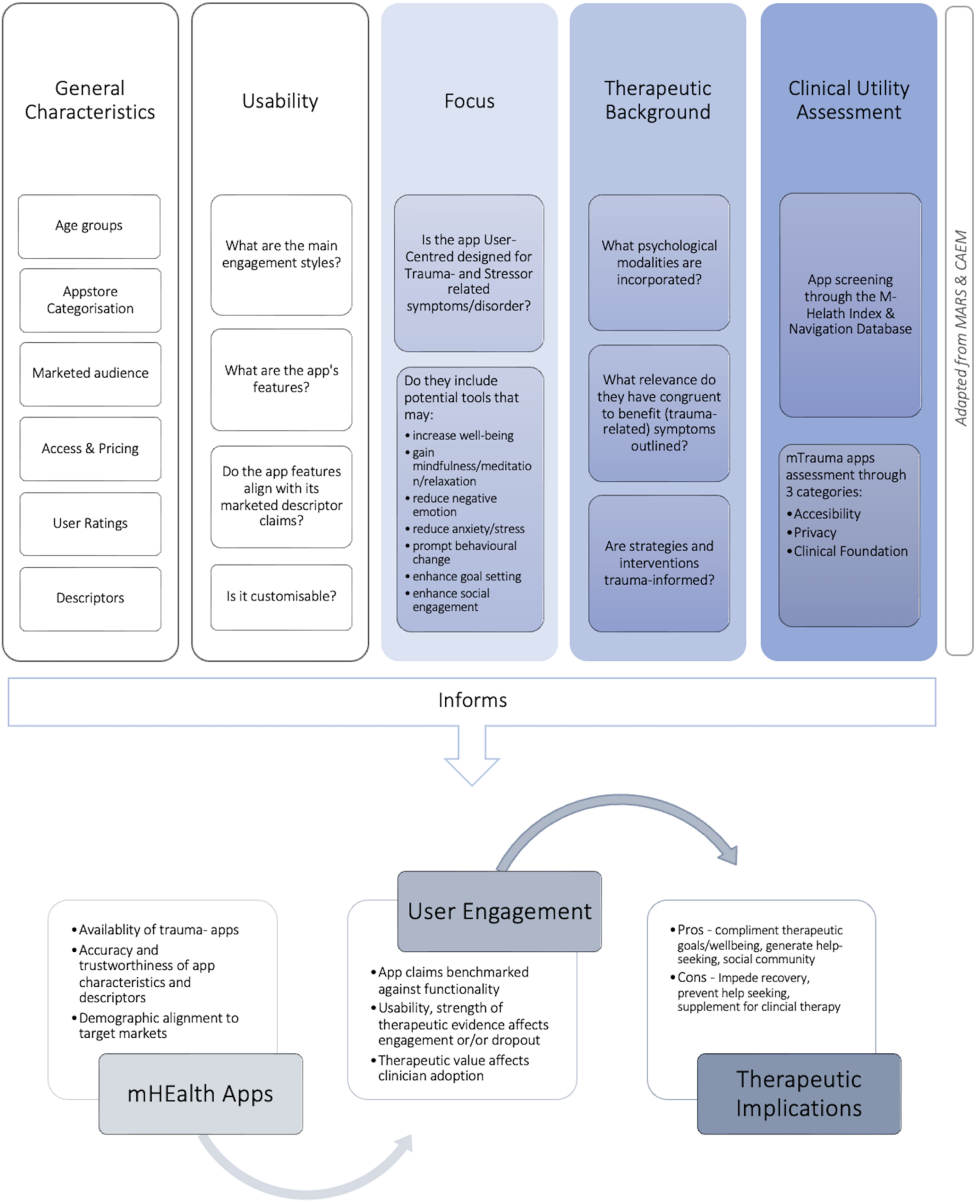
Ting & McLachlan (2022) mTrauma app informatics evaluation framework.

The first section presented in our mTrauma App Informatics Evaluation Conceptual Framework displayed in Fig. 4 extends across five domains ((1) General Characteristics, (2) Usability, (3) Focus, (4) Therapeutic Background, and (5) Clinical Utility Assessment) in which are discussed accordingly. Facilitated through a comprehensive informatics descriptive approach, the authors identified available options of trauma-related apps that are publicly accessible and discussed their mental health purpose based on marketed descriptors, intended target audience, and characteristics.

A total of 81 apps sufficed the inclusion criterion. Based on the observations provided by the apps’ general characteristics, the inclusions of target markets for trauma-related mhealth tools have expanded. Previous research demonstrated trauma-informed mobile tools were monopolised in their delivery and designed specifically to support military servicemen, combat soldiers and veterans (Kuhn et al., 2018; Erbes et al., 2014; Montena et al., 2022; Owen et al., 2018). Similarly levelled, a reasonable number of apps now cater to frontline personnel in both public and private sector job profiles with high propensities of trauma exposure to encourage early detection, symptom management and accelerated access to clinical support (Alexopoulos, Hudson & Otenigbagbe, 2020; van der Meer et al., 2017). Despite the slight expansion of including wider markets, only three apps (*n* = 3, 3.7%) targeted specified cohorts. *Couple HOPES* categorised under ‘lifestyle employed PTSD-informed strategies to improve their symptoms and relationships accompanied by a coach. *TraumaMAMAs* was designed to facilitate mothers with histories of trauma applying curated tools, resources, and access to periodical expert advice. *Trauma Healing Yoga* was the only app that specifically designed for sexually traumatised women. Studies of somatic body-based therapies have argued the benefits of integrating body movements in trauma healing (Justice, Brems & Ehlers, 2018; Macy et al., 2018; Gulden & Jennings, 2016). Considering somatic-based apps are at an early stage of the development life cycle, it would be interesting to examine user engagement and uptake in comparison to passive-engagement apps to determine their therapeutic benefits. Although there is an expansion to target wider markets, many apps currently available undertake a mass-audience approach but lack a specified curation that would be ideal for individualised trauma types including intimate partner violence, sexual abuse, social phobia, gender and or identity discrimination, natural disasters, refugees and immigrants, school shootings or mass-trauma (such as the COVID-19 pandemic and the Russian-Ukrainian crisis). User engagement and clinicians are confronted with usability uptake due to the plethora of apps available and their generalised approach. Understanding there is a global increase across communities of traumatic events, there is a need to expand the focus of mtrauma apps to include its pertinence for victims of domestic violence, discrimination, transgenerational trauma and mass trauma (Ting & McLachlan, 2022).

Most apps were marketed for the ages 4-years and above, with the highest target audience allocated to adolescents, children, parents, caregivers alongside clinicians and clients. Do these findings indicate a supply that meets a demand, one in which individuals are being diagnosed with trauma disorders at a younger age or are children and adolescents encountering more traumatic experiences in current society? The literature suggested two thirds of children experience one traumatic event by the age of 16 (Substance Abuse & Mental Health Services Administration, 2022), and at least 50% of children across three continents (Asia, Africa, and North America) had experienced violence in 2018 alone which corresponds to more than one billion children victimised worldwide (van Der Kolk, Ford & Spinazzola, 2019). An estimated 3% to 15% of girls and 1% to 6% of boys with histories of trauma will develop PTSD (National Center for PTSD, 2022). Adverse global events, such as the forced isolation of COVID-19, disconnection caused by school closures, and disruption of normality have heightened various stress reactions in younger cohorts (Jiao et al., 2020). Following the global pandemic, the invasion of Ukraine seen millions of children affected by the war and more than 90,000 displaced across care facilities, with half of them being living with disabilities (Schlein, 2022). The lengthy COVID-19 pandemic may have highlighted the benefits of utilising mhealth platforms as a treatment companion. Practitioners require careful considerations in examining background factors before apps are concluded for safe usage with patients (Owings-Fonner, 2020). Trauma-related symptoms are deemed high risks in clinical conditions. The efficacy for trauma-disorders adopting mobile application interventions were proven to enhance treatment and reduce symptoms but centralised in trials with adult participants (Wickersham et al., 2019; Olff, 2015; Goreis et al., 2020) or accompanied by a clinician. Although few apps in this study displayed descriptors stating user engagement may benefit well-being and not to be mistaken as medical advice, there are no strict barriers to prevent children or teens in accessing the app’s content without parental guidance. The usefulness of applications for children, preadolescents, and adolescents with mental health issues is presently not sufficiently supported by research (Grist, Porter & Stallard, 2017). It is urgently necessary to conduct methodologically sound research studies assessing the safety, efficacy, and usefulness of mHealth applications given the volume and rate at which they are being introduced on app stores without the disclaimer of parental guidance. Considering that many applications are ‘free’ to download, trauma-related apps require additional supporting evidence to justify it is safe for ages 4+ to consult with accompaniment to defend its marketed approach. To detect and minimise unforeseen risks from app engagement, the appropriate ‘age’ marketed by tech developers should be co-designed with young people, and where applicable for children, their parents, alongside psychological expertise.

Observing mhealth marketing approaches and user ratings enhances insight to their established value to target markets and informs use engagement. Close to 90% of apps were complimentary to download but with considerations that included in-app purchases were not always explicitly mentioned in its front-matter content and prompted users at a later stage. Although this marketing ‘hook’ attracts initial user uptake, it may affect the ‘drop out’ rate of continuous usage. mHealth apps categorised as ‘health and fitness’ under the regulatory frameworks in the free-app market lack coherent risk-assessment models (Terry & Gunter, 2018). From a mental health perspective, there are considerations as to how the lack of access (*e.g*., due to financial restrictions) may heighten distress in individuals that have adopted substantial reliance on these supplementary tools. Only a few apps provided the transparency of short-term trials that required up-front credit card details to gain access and post-trial membership costs. Eleven apps required payment which ranged from $1.49 to $14.99 Australian dollars, but despite their affordable range, the cost may only include access to the app whereby additional subscriptions may apply. Seeing as to how customers may feel when an app is portrayed as ‘free’ which later requires a membership or payment, users may feel a need to search for a new suitable application. This may lead to a loss of continuity of care, even it is an app. Apps awarded with 3* to 5* were affected by mood tracking or apps with built in journals but ratings for paid apps were largely scarce. Although ratings are present, and potentially influencing users when selecting an app, their short marketing descriptions rarely provide sufficient information on how suitable an app is (Neary & Schueller, 2018). There is a need for future studies to illuminate the causal effects of free *vs*. paid m-health apps in relation to user engagement and understanding the qualities users based their ratings on. Additionally, for a general audience discontinuing an app or purchasing an app that an individual comes to dislikes may not be as harmful as the risks it would pose to an individual with mental health challenges and their reasoning behind engagement with a mental health app. A push to regulatory frameworks may benefit applications marketed under ‘health & fitness’ to include a criterion that outlines its therapeutic foundations, treatment options, tools and services that require payment in which allows a user (or accompanied by a parent/clinician) to make an informed decision that may minimise risk and distress.

We examined the applications tools and functionality, extracted psychological foundations present or implied within their framework designs, and evaluated their therapeutic efficacy. The most utilized therapeutic modalities were CBT and PTSD-informed. However, we observed a rise of apps utilizing eye-movement desensitization and reprocessing (EMDR) and emotional freedom tapping. EMDR, a psychotherapy modality originally designed to treat post-traumatic stress disorder, has been further identified as complimentary treatment beneficial for trauma-related psychopathology including affective disorders (Perlini et al., 2020; Adams, Ohlsen & Wood, 2020; Karadag, Gokcen & Sarp, 2020; Beer, 2018). The efficacy of applying EMDR in early interventions shortly after traumatic events displayed reduction in symptoms and prevents exacerbation of symptoms (Shapiro & Maxfield, 2019). Emotional freedom technique (EFT) is a therapy that uses a sequence of combined tapping techniques, acupressure (massaging acupuncture points) and reciting activating phrases based on cognitive behavioural therapy of self-acceptance and affirmation (Sebastian & Nelms, 2017; Motta, 2020). It has been demonstrated that EFT (alike other somatic therapies) can desensitize clients without necessitating the need to participate in vocally reliving traumatic events (Stapleton, 2019, P. 36). Introducing the inclusion of sensory and body-based techniques within trauma-related mobile apps are promising as it expands alternative options where traditional cognitive based therapies are not preferred.

Apps that included a sub-section that were useful to support symptom management were dominated by journal and mood tracking apps. Others observed mindfulness, meditation, art therapy, positive psychology, sensory and image identification, with two apps embodying somatic therapies including yoga therapy and tension release. The majority of apps contained tools proven by previous studies to be useful in treating symptoms related to or as a comorbid condition of trauma in the provision of psychoeducation, training activities, guided sessions, and symptom management (Wickersham et al., 2019; Drissi et al., 2019; Gindidis, Stewart & Roodenburg, 2019). Some apps included advice, links to services, and access to providers that may enhance the challenges faced by clinicians to provide care in a timely manner (Litz & Kerig, 2019). Studies have demonstrated the benefits of mental health-related self-tests using mobile apps as an ideal platform for large-scale dissemination and reducing the burden on healthcare providers provided. Hence, such platforms are often utilized in connection with professional guidance (Shang et al., 2019). Self-assessments were present in a few apps but did not outline if users received a full assessment or how users may approach their results without the accompaniment of a qualified health professional.

Only 17 (21%) out of 81 trauma-apps are registered on the mental health index and navigation database indicates that many apps are not being recognized as having official accreditation to support the source of information and associated support for mental health. This further outlines the complexities that clinicians and app users face in seeking reliable, effective, and safe trauma apps for therapeutic utility and integration. In examining the apps registered on the MIND website, the overall trauma-app’s combined rating arrived at a quality score of 75%. However, this score may be insufficient for clinicians recommending app uptake when considering seven apps (41%) do not specify data proprietorship, which poses security concerns, and 47% of apps (*n* = 8) do not allow exporting data for clients. The latter thus does not support seamless integration of patient data for ongoing therapy management across more than one provider. Accessibility, security, and privacy concerns are significant in directing clinical recommendations (O’Loughlin et al., 2019; Rowland et al., 2020). Tech developers should prioritize transparently displaying these details in app descriptors prior to downloading the app. This is especially pertinent when considering that mHealth apps have target consumers may have additional vulnerabilities (Sax, Helberger & Bol, 2018). Positively, many registered apps (*n* = 16, 94%) include a transparent privacy policy, relevant content and performs to their marketing claims. Criteria 8 proves useful for both clinicians and clients in therapy as conditions are specified. However, the majority that did so (*n* = 10, 59%) was largely focused on PTSD, followed by other comorbidities (mood, sleep, stress, and anxiety), reiterating the lack of focus that attends to other trauma and stressor-related disorders. Overall, these results suggest that there is a need for greater attention on assessing the quality and reliability of mental health apps, its audiences and target marketing, to ensure that users are provided with accurate and effective information and support for their mental health needs.

### Limitations

This study has several limitations. Firstly, the authors retrieved apps from only the App Store that were accessible in Australia and were in English. Next, our results may have omitted suitable trauma-related apps which did not originate through our search strategy and key terms. This may be attributed to App Store’s in-built embedded algorithmic search engines which posits unidentified apps that may be eligible if sourced through an alternative platform such as ‘Google Play’. Thirdly, due to the fast-paced turn-over of mobile applications, some apps may have been removed, updated, or changed at the time readers review our study. Fourthly, we analyzed apps using a cross-adaptation of the MARS and CAEM evaluation, based on app descriptors and sourced additional information through developer websites. Since the apps were not downloaded, there may be elements pertaining to their tools, design, and other caveats that were absent from our analysis. Further enquiry may adopt more novel app evaluation instruments as mhealth apps grow in popularity. In order to determine quality assessment with homogeneity, only 17 apps out of 81 apps were available from the M-Heath Index Database. Lastly, authors attempted to source additional information from developer websites to discuss therapeutic validity using evidence-based results and clinical trials. This attempt was not successful as many apps did not have a developer website and those that did, did not offer beneficial information that indicated clinical trials were conducted or provided results from user engagement. Thus, the authors cannot provide confidence that apps were certified through evidenced-based research due to the lack of publicly available information. A plausible hypothesis for the lack of quality options of clinical endorsement perhaps indicates their invalid position as a supplementary mhealth tool.

## Conclusions

Through an informatics analysis study, this article identified the availability of trauma- and stressor-related mobile applications, its tools, functionality, and psychological underpinnings and discussed their therapeutic value. Additionally, this study further assessed the therapeutic utility of mTrauma apps registered from the MIND that determined accessibility, privacy and clinical foundation with a rating score of 75%. We observed the introduction of newer trauma-informed psychotherapeutic modalities available in the App Store alongside the inclusion of wider target markets. Aside from its specified models of design for veterans and PTSD, currently available apps employ a jack-of-all-trades approach utilized for general trauma symptoms or through associated comorbid conditions. The complexities of trauma-related symptoms and disorders require curating value-added mobile applications that are designed specifically for sociological traumatic histories such as sexual abuse, long covid, racism, work-place incidents, gender-discrimination, grief, and violence.

## Supplemental Information

10.7717/peerj.15366/supp-1 Supplemental Information 1 Raw Data. The search strategy, apps extracted, duplicates removed and app characteristics.

## Additional Information and Declarations

### Data Availability

The following information was supplied regarding data availability: Raw data are available as a Supplemental File.
